# Trends in weekly reported net use by children during and after rainy season in central Tanzania

**DOI:** 10.1186/1475-2875-11-218

**Published:** 2012-07-02

**Authors:** Hannah Koenker, Beatriz Munoz, Marc Boulay, Harran Mkocha, Joshua Levens, Sheila K West, Matthew Lynch

**Affiliations:** 1 Johns Hopkins University Bloomberg School of Public Health Center for Communication Programs, Baltimore, MD, USA; 2 Dana Center for Preventive Ophthalmology, The Wilmer Institute, The Johns Hopkins University, Baltimore, MD, USA; 3 Kongwa Trachoma Project, Kongwa, Tanzania

## Abstract

**Background:**

The use of long-lasting insecticidal nets (LLINs) is one of the principal interventions to prevent malaria in young children, reducing episodes of malaria by 50% and child deaths by one fifth. Prioritizing young children for net use is important to achieve mortality reductions, particularly during transmission seasons.

**Methods:**

Households were followed up weekly from January through June 2009 to track net use among children under seven under as well as caretakers. Net use rates for children and caretakers in net-owning households were calculated by dividing the number of person-weeks of net use by the number of person-weeks of follow-up. Use was stratified by age of the child or caretaker status. Determinants of ownership and of use were assessed using multivariate models.

**Results:**

Overall, 60.1% of the households reported owning a bed net at least once during the study period. Among net owners, use rates remained high during and after the rainy season. Rates of use per person-week decreased as the age of the child rose from 0 to six years old; at ages 0–23 months and 24–35 months use rates per person-week were 0.93 and 0.92 respectively during the study period, while for children ages 3 and 4 use rates per person-week were 0.86 and 0.80. For children ages 5–6 person-week ratios dropped to 0.55. This represents an incidence rate ratio of 1.67 for children ages 0–23 months compared to children aged 5–6. Caretakers had use rates similar to those of children age 0–35 months. Having fewer children under age seven in the household also appeared to positively impact net use rates for individual children.

**Conclusions:**

In this area of Tanzania, net use is very high among net-owning households, with no variability either at the beginning or end of the rainy season high transmission period. The youngest children are prioritized for sleeping under the net and caretakers also have high rates of use. Given the high use rates, increasing the number of nets available in the household is likely to boost use rates by older children.

## Background

The use of long-lasting insecticidal nets (LLINs) is one of the principal interventions to prevent malaria in young children, reducing episodes of malaria by 50% and child deaths by one fifth [1]. A 2007 review of seven demographic surveillance sites in Africa found that the median age at death of children under 15 ranged from 1.01 to 1.65 years among the different sites [2]. Prioritizing young children for net use is therefore important to achieve mortality reductions, particularly in high transmission zones.

While some studies have shown that adults are often prioritized for net use when nets are scarce [3,4], more recent studies appear to show the impact of ongoing net distributions and communication interventions, with younger children (often sleeping with their mother) experiencing higher rates of net use [5] or being prioritized for the best nets [6]. Currently, the recommended indicator for measuring net use is ‘net use the previous night’, which tends to vary according to whether the survey was conducted in rainy or dry season [3,7-15], but cross-sectional surveys cannot measure consistent net use over time. The aim of this study was to measure patterns of net use within households before, during and after the rainy season in a rural area of Tanzania, using short surveys administered weekly. The study was done in conjunction with the PRET (Partnership for the Rapid Elimination of Trachoma) Plus study [16] in 2009. The PRET Plus study was a 6-month, population-based, prospective cohort study designed to evaluate the ancillary benefits after mass treatment with azithromycin for trachoma with respect to the incidence and prevalence of malaria (*Plasmodium falciparum*), relapsing fever, invasive diarrhoeal disease, acute respiratory illnesses (ARI) and sexually transmitted diseases.

At the time of the survey, neither the Tanzania under-five catch-up campaign nor the universal coverage campaign had taken place. Nationally, ownership of any type of net in Tanzania in 2007–8 was 56% [17], with subsidized distribution of LLINs occurring through the Tanzania National Voucher Scheme to pregnant women and infants, a system, which has been described in detail elsewhere [18-20], and at full price through the commercial sector.

## Methods

The PRET Plus study was conducted in Kongwa district of Dodoma Region, located in rural central Tanzania. The total population of Kongwa district is approximately 248,656 and most villagers are herders or subsistence farmers. The regional climate is that of a highland plateau with semi-arid conditions where access to water is poor. For study purposes, the communities represented the smallest population unit for which health services are organized and trachoma control programs are implemented. Tanzanian communities are geographically distinct sub-villages, averaging 1,500 persons, including an estimated 200–380 persons aged less than five years. The PRET Plus study methodology is described elsewhere [16].

The PRET Plus study was conducted from January 12 through July 21, 2009, during an uncharacteristically dry period in the region where the rainfall total for the year (331.2 mm) was 60% of expected, and occurred mainly in the months of February and March. The study included eight communities located in the Kongwa District of Tanzania. All communities were located in close proximity such that environmental conditions were similar. Prior to study initiation, community leadership provided consent to the overall community participation in the study.

One month prior to the beginning of the surveillance a complete census of the eight communities was carried out. All households with at least one child under the age of five were eligible for the study. In each community 130 eligible households were selected at random, and within each selected household a child under the age of five was randomly selected to serve as the index child for surveillance purposes. A total of 1036 households were selected. All random selections were performed in advance of the baseline assessment by a computer-generated algorithm using a stream of uniformly distributed random numbers.

Longitudinal surveillance of the 1036 households was carried out through weekly visits. For each study week, among the residents of households that reported owning a bed net that week, the proportion of children sleeping under nets the previous night was calculated, stratified by age of the child. The data was then assessed for overlap with the rainy season, which lasted from mid-February through the end of March. The characteristics associated with households ever reporting owning a bed net were examined using logistic models for both the bivariate and multivariate analyses, the Generalized Estimated Equation (GEE) approach was used to account for the clustering effect at the community level.

To investigate the household/child characteristics associated with the child sleeping under a bed net, all children under seven years of age from families reporting owning a bed net during the weekly surveillance were included. While studies generally report bed net use for children under five, children under seven were included to capture any patterns in use related to number of older siblings. The usage rate was estimated as number of weeks reporting that the child slept under a bed net divided by the number of follow-up weeks for that child. Incidence rate ratios and 95% confidence intervals were used to describe the association between putative risk factors and bed net use. Models for count data using a negative binomial link function with the numbers of follow-up weeks as the offset variable were implemented to estimate the incidence rate ratios. Standard errors of the estimates were corrected using the GEE approach to account for the clustering effect at the household level.

## Results

**Figure 1:**
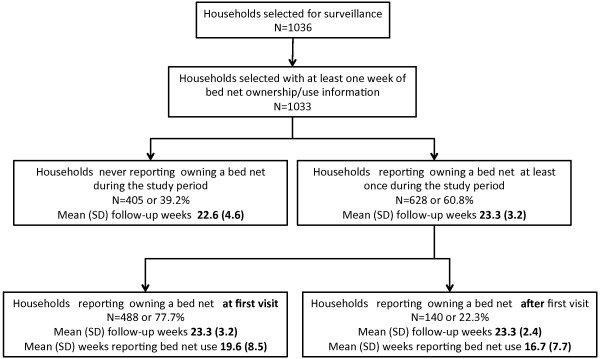
Study households.

A total of 1,036 households were selected for surveillance; 1,033 households had at least one week of bed net ownership and use information (Figure 1). Out of these households 39.2% (n = 405) never reported owning a bed net during the study period. Of the remaining 60.8% (n = 628) households that did report owning a bed net at least once during the study period, 77.7% (n = 488) reported owning a bed net at first visit and 22.3% (n = 140) reported owning a net only after the first visit. The mean follow-up weeks for non-net-owning households was 22.6 weeks; for net-owning households the mean was 23.3 follow-up weeks. Households reporting ownership at first visit had a mean of 19.6 weeks where they reported bed net use; households reporting ownership after the first visit had a mean of 16.7 weeks of net use.

Bed net ownership and use of bed nets were examined against household characteristics in a univariate model. Bed net ownership was significantly associated with younger head of household (mean age for non-owners was 40.9 years, while mean age for net owning households was 37.1), and with farther distance to a water source during the dry season (Table 1). Household size, number of children under seven, years of education of head of household, whether the house had a latrine, and whether the head of household was male were not associated with ownership of nets.

**Table 1:** Household characteristics by bed net ownership

**Characteristic**	**Did not own a bed net at any time over the study period**	**Reporting owning a bed net at least once time over the study period**	p-value*
N	405	628	
Distance to the water source dry season			0.05**
% < 30 minutes	33.4	23.3	
% 30–60 minutes	35.6	30.4	
% > 60 minutes	30.9	46.3	
% House has a latrine	67.6	71.8	0.27
% Male head of household	68.4	73.7	0.11
Age of head of household (mean (sd))	40.9 (14.2)	37.1 (11.7)	<0.001
Years of education head of household	3.6 (3.5)	3.8 (3.5)	0.34
Household size (mean(sd))	5.8 (2.2)	5.7 (2.1)	0.49
# children under 7 years of age	2.0 (1.9)	2.0 (1.8)	0.76

*Adjusted to account for clustering at village level; ** test for trend.

In the multivariate model for bed net ownership (Table 2), when controlling for distance from water, the odds of owning a bed net increased by 12% for each five-year decrease in the age of the head of household. When controlling for age of the head of household, the trend remained for increased likelihood of bed net ownership for those households located further away from dry-season water sources. However, the only statistically significant difference in net ownership was between households located 30–60 minutes and those located more than an hour from a dry-season water source.

**Table 2:** Household characteristics associated with bed net ownership. Multivariate model

**Characteristic**	**Odds Ratio**	95% Confidence Interval*
**Distance to the water source dry season**		
< 30 minutes	1.00	
30-60 minutes	1.21	(0.61 – 2.43)
> 60 minutes	2.17	(0.97 – 4.87)
Male head of household	1.22	(0.88 – 1.71)
Younger age of head of household (per 5 years decrease)	1.12	(1.06 – 1.18)

*Adjusted to account for clustering at village level and test for trend 0.053.

Bed net use was also tracked for each household (Table 3). For children under seven in net-owning households, person-weeks of net use were calculated over the study period. The age of the child, the number of children under seven in the household, the age of head of household, and (marginally) education level of head of household were significant factors in the rates of bed net use per person-week.

**Table 3:** Rates of bed net use in children under seven by child/household characteristics

**Characteristic**	**Person-weeks**	**# events**	**Rate/per person-week**	Incidence rate ratio (95% CI)*
**CHILD**				
**Gender**				
Male	11246	9201	0.82	1.01 (0.96 – 1.06)
Female	12113	9807	0.81	1.00
**Age**				
0-23 months	7374	6845	0.93	1.67 (1.52 – 1.85)
24-35 months	3615	3323	0.92	1.67 (1.51 – 1.85)
3	3927	3359	0.86	1.54 (1.39 – 1.71)
4	3247	2607	0.80	1.44 (1.29 – 1.62)
5-6	5196	2874	0.55	1.00
**HOUSEHOLD**				
**Number of children under 7 in the HH**				
1	3549	3443	0.97	1.67 (1.34 – 2.09)
2	10167	8552	0.84	1.46 (1.16 – 1.82)
3	7650	5809	0.76	1.32 (1.05 – 1.66)
4 or more	1993	1204	0.60	1.00
**Number of HH members 7 or older**				
1-2	7200	6314	0.88	1.18 (1.03 – 1.35)
3-4	8933	7071	0.79	1.06 (0.92 – 1.22)
5-6	5305	4167	0.78	1.05 (0.91 – 1.22)
>6	1921	1456	0.76	1.00
**Age head of household**				
17-30	7207	6282	0.87	1.12 (1.05 – 1.20)
30-39	8302	6526	0.77	1.00 (0.93 – 1.08)
40 or older	7850	6200	0.79	1.00
**Gender of head of household**				
Male	17907	14484	0.81	1.00 (0.94 – 1.07)
Female	5380	4452	0.83	1.00
**Education head of household**				
None	10843	8521	0.79	0.94 (0.88 -1.00)
1-6 years	1690	1503	0.89	1.04 (0.95 – 1.15)
7 or more years	10826	8984	0.83	1.00
**Distance to water source dry season**				
<30 minutes	4448	3737	0.84	1.02 (0.94 – 1.10)
30-60 minutes	6777	5478	0.81	1.00 (0.94 – 1.07)
>60	12011	9700	0.81	1.00
**House has a latrine**				
No	6119	5034	0.82	1.00
Yes	16865	13641	0.81	0.98 (0.92 – 1.04)
Overall	23359	19008	0.814	---------------

*Fitted using a negative binomial distribution with offset equal to the logarithm of number of follow-up weeks. The GEE approach was used to account for clustering at the household level.

Rates of use per person-week decreased as the age of the child rose from 0 to six years old; at ages 0–23 months and 24–35 months use rates per person-week were 0.93 and 0.92 respectively during the study period, while for children ages 3 and 4 use rates per person-week were 0.86 and 0.80. For children ages 5–6 the rate dropped to 0.55. This represents an incidence rate ratio of 1.67 for children ages 0–23 months compared to children aged 5–6. Caretakers’ incidence rates were 0.94 per person-week. Ninety-nine percent of caretakers were females.

**Figure 2:**
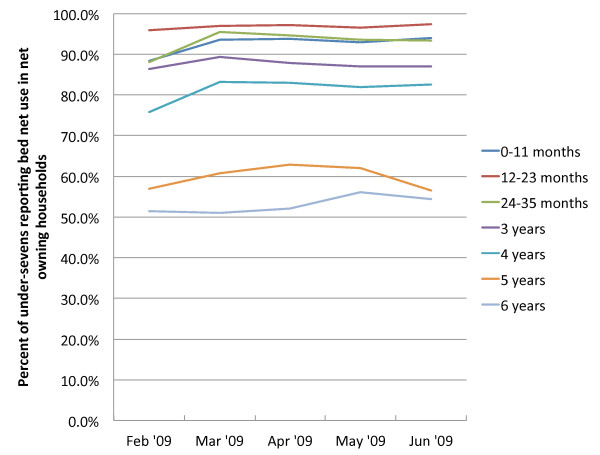
Bed net use in children residents of households reporting owning a bed net, by age.

**Figure 3:**
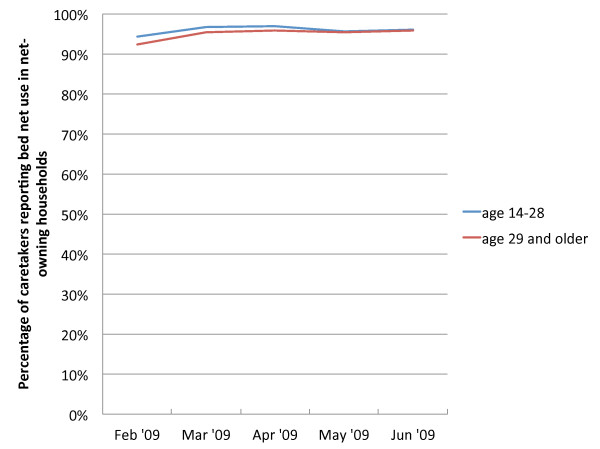
**Bed net use among caretakers of children residents of households reporting bed net ownership, by age.** *99% of caretakers were female.

Overall, child use rates per person-week were 0.81, or 19,008 events (weeks in which the child was reported to have slept under a net) out of a total of 23,359 person-weeks of follow-up. Figure 2 describes the trends in use by children over the study period. Children 12–24 months maintained use rates of over 95% between February and June. Use of nets by children under 12 months and children age 24–35 months rose slightly from 86% in the first full month of follow-up and remained steady at around 93% until June. Use rates for older children maintained similar trends over time, remaining steady throughout the study period. Figure 3 finds a similarly stable trend in net use for caretakers, whose use rates match those of the children 12–24 months, with no significant difference in use between caretakers under 29 and those aged 29 or older.

Having fewer children under seven in the household also appeared to impact net use rates for individual children. In households where there was only one child under seven years, the children had a 67% greater likelihood of using the net compared to households where there were four or more children. Children in households with only 2 or 3 children also had an increased likelihood of net use (46% and 32%, respectively) compared to those with four or more children.

Compared to heads of household aged 30 or more, heads of household who were 17–30 years old were 12% more likely to have their children sleeping under a net. Education of head of household was borderline significant, with those reporting no education slightly less likely (0.94 incidence rate ratio) to have their child sleeping under a net, compared to those with some primary or secondary education.

The rate of use per person-week did not differ significantly for male vs female children, gender of head of household, whether the house had a latrine, or for distance to water source during the dry season.

Within households that owned a net, household and individual characteristics were independently associated with children sleeping under a net (Table 4). The multivariate model includes child age, number of children under seven in the household, education of the head of household, and age of the head of household. When controlling for other variables, each of these factors remains significant. The incidence rate of bed net use for children age 0–35 months is 1.61 times that of children aged 5–6; children age three and four had incidence rates 1.50 and 1.40 times that of the older group. The number of children under seven in the household also showed a negative association with bed net use rates. Age and education of the head of household remain significant; children living with a household head with some education have a 7% increase in incidence rate than do children in households where the head of household has no education. Likewise, children in households with heads of households under 30 years of age have an 8% increase in bed net use incidence rate compared to children living with heads of household over 30. Table 5 describes characteristics by village; net ownership rates ranged from 31% to 86% among villages; use rates by children under seven in net-owning households ranged from 69% to 94%. Percentage of households living more than 30 minutes from a dry-season water source ranged from 37% to 100%; size of the villages, latrine ownership and years of education of the head of household were largely similar.

**Table 4:** Characteristics independently associated with children sleeping under a bed net in households reporting owning a bed net. Multivariate model*

**Characteristic**	**Incidence rate ratio**	**95% Confidence Interval**
**Child’s age**		
0-35 months	1.61	1.46 – 1.78
3 years	1.50	1.35 – 1.66
4 years	1.40	1.25 – 1.57
5-6 years	1.00	
**Number of children under 7 in the household**		
1	1.48	1.18 – 1.86
2	1.40	1.10 – 1.78
3	1.31	1.02 – 1.67
4 or more	1.00	
**Education head of the household**		
None	1.00	
Some	1.07	1.00 – 1.14
**Age head of household**		
17- 30 years	1.08	1.01 – 1.15
Older than 30 years	1.00	

*Fitted using a negative binomial distribution with offset equal to the logarithm of number of follow-up weeks. The GEE approach was used to account for clustering at the household level.

**Table 5:** Characteristics of the 8 communities

**Village ID**	**Total Population**	**% > 30 minutes from water**	**% houses with latrine**	**Mean years of education head of household**	**% Net Ownership**	**% Net use**
1	1726	56.1	77.7	3.4	62	82
2	1323	37.2	76.6	4.3	68	85
3	1528	100.0	60.9	2.4	61	79
4	1710	100.0	81.1	3.3	81	94
5	1714	77.2	69.2	3.4	52	72
6	1938	96.7	61.2	2.7	86	95
7	1666	67.0	72.9	3.1	41	69
8	1292	53.8	70.4	4.0	31	69
**Mean (SE)**	**1612 ± 77**	**73.5 ± 8.5**	**71.3 ± 2.6**	**3.3 ± 0.22**	**60 ± 6.6**	**81 ± 3.6**

## Discussion

In this study, 60.8% of households reported having a bed net, slightly higher than national estimates from the same period [17]. The continued high rates of use over the study period are indicative of the importance these households place on nets. The rainy season was quite short during early 2009, lasting from February to mid-March only, but rates of net use remained high through June. Behaviour change communication (BCC) activities in Kongwa were fairly limited in the community prior to and during the study period. However, ongoing BCC to support the TNVS had been occurring since 2004 and national radio spots on malaria prevention were being aired periodically. While the study design did not examine exposure to BCC messages as determinants of net use, it is probable that most households had encountered a significant amount of information and bed net use promotion from various sources over the previous several years, encouraging infants and pregnant women to be prioritized for net use. Previous studies in Tanzania have shown that young children tend to sleep under the ‘best’ nets [6]. Further studies should explore the links between high rates of bed net use and exposure to BCC messaging to ascertain the impact of BCC messages on these types of preventive behaviours.

Within the households, use of insecticide-treated bed nets contributes to protection from mosquito bites for members of the household who do not have a net to sleep under [21-25] as well as for other members of the community [26-31]. Estimates of thresholds for community-level protection range from 35-65% from modeling [32] to 50% as described in Kenya [26]. Six of the eight villages had ownership rates over 50% (Table 5), indicating that this level of spatial coverage could be providing significant protection for the entire community, even those households without nets.

The fact that the households were visited weekly and asked about bed net use may have resulted in over estimation of use and is likely to have contributed to a Hawthorne effect, but the extent to which this is true cannot be known. The weekly visits also covered symptoms of malaria, fever, and diarrhoea so there was a focus on other factors that were being studied as well. This study demonstrates that in this area of Tanzania, net use remains high among net-owning populations both during and beyond rainy season.

The Tanzania National Voucher Scheme was ongoing prior to and during the study period, and we posit that younger heads of household were more likely to have infants and/or pregnant women in their households who were thus eligible for a voucher than were the older heads of household. However, there was no readily available information in Kongwa on the voucher scheme during the time of this study.

Distance from water during the dry season was a significant determinant for net ownership, but not for use. This finding appears to be counterintuitive, as households located close to water sources might be expected to have an increased number of nuisance-biting mosquitoes and, therefore, be more motivated to acquire nets. However, water sources in Kongwa are deep wells that need either windmills or generators to pull water up. During rainy season, there is almost universal access to seasonal small shallow lakes, which were not asked about. Since distance to water is associated with trachoma and other indices of poverty, it suggests that poorer households may be more likely to have nets. In part this might be explained by poorer access to treatment should disease occur, thus enhanced stimulus to own nets, but this would need to be formally tested.

No data was collected on the total number of nets in the household; however, the 2007–2008 Tanzania HIV and Malaria Indicator Survey conducted 12 months prior reported that 44% of households in Dodoma region owned at least one net of any type, and 28% owned an ITN, with an average number of 0.7 nets per household [17]. Owning only one net would explain why children in households where they are the only child or have only one other sibling are more likely to have slept under a net over the study period, whereas there was no difference for children with two to five or more siblings. The youngest children appear to be prioritized for net use over older siblings, in line with behaviour change communication efforts over the past decade that promote net use for young children, particularly infants. This may also indicate that mothers are keeping their infants with them under the net until they are weaned or a subsequent child is born, supported by the high use rates by caretakers. The recent under-five catch-up campaign carried out between March 2009 and May 2010 provided an LLIN to each child under five [33]; the universal coverage campaign provided additional nets to households in 2010 and 2011. If coverage is not maintained, however, through an additional delivery channel(s), net ownership is likely to decline over time as families grow and nets wear out; households would then once again be faced with the choice of which household members to prioritize for net use.

### Limitations

There were some limitations to our study. We did not verify the existence of a net, and so some households may have reported net use due to social desirability bias. Of note, though, at the outset, of the 140 households that reported owning a net after the first visit, several households told the interviewer they thought if they reported no net that one would be provided by the study. Thus, there was more likely some negative incentive to report net use, which corrected itself over time. Partial use of the nets was not captured, that is, if the nets were not used in early evening due to the day’s heat, but were used as the night wore on. This would lead to less protection, but reported use in the previous night.

The strength of the study is the large sample that was visited weekly to determine incidence rates, and that these weeks were over the malaria season in Kongwa.

## Conclusions

In this area of Tanzania, net use was very high and consistent among net-owning households, with no variability either at the beginning or end of the rainy season high transmission period. The youngest children were more likely to sleep under the net and caretakers also have high rates of use. Given the high use rates, increasing the number of nets available in the household is likely to boost use rates by older children.

## Competing interests

The authors declare that they have no competing interests.

## Authors’ contributions

HK designed the study and drafted the manuscript, BM conducted the analysis, JL and HM coordinated the fieldwork, MB performed additional analysis, SW provided funds and field support, and ML, SW and BM provided comments on the manuscript. All authors read and approved the final manuscript.
